# Rapid, quantitative lateral flow immunoassay using polystyrene-gold composite nanoparticles and CIELAB analysis for on-site detection of *Listeria monocytogenes* in food samples

**DOI:** 10.1007/s00604-025-07795-6

**Published:** 2025-12-28

**Authors:** Zhijian Wang, Ya-Ching Yu, Xiaoyu Ji, Yixuan Ding, Amanda J. Deering, George T.-C. Chiu, Jan P. Allebach, Lia A. Stanciu

**Affiliations:** 1 Weldon School of Biomedical Engineering, Purdue University, 610 Purdue Mall, IN 47907 West Lafayette, USA; 2 School of Materials Engineering, Purdue University, 701 West Stadium Ave., IN 47907 West Lafayette, USA; 3 School of Electrical and Computer Engineering, Purdue University, 465 Northwestern Ave., IN 47907 West Lafayette, USA; 4 School of Mechanical Engineering, Purdue University, 585 Purdue Mall, IN 47907 West Lafayette, USA; 5 Department of Food Science, Purdue University, 745 Agriculture Mall Dr., IN 7907 West Lafayette, USA; 6 Bindley Bioscience Center, Purdue University, 1203 W State St., IN 47907 West Lafayette, USA; 7 Department of Materials Engineering, Purdue University, IN 47907 West Lafayette, USA

**Keywords:** Nanoparticle, Antibody, Listeria monocytogenes, Lateral flow biosensor, Inkjet printing, CIELAB, Image analysis, Colorimetric detection

## Abstract

Sensitive, enrichment-free detection of *Listeria monocytogenes* in food matrices is still challenging. Traditional lateral flow immunoassays (LFIA) come with limited sensitivity and often require lengthy pre-enrichment steps, which is a roadblock for rapid and on-site testing. In this work, a novel LFIA platform was developed that uses surface-modified polystyrene-gold nanoparticle composites (Ps-AuNPs) as enhanced colorimetric labels. The resulting signal enhancement enables the detection of *L. monocytogenes* without pre-enrichment steps. The assay also uses *International Commission on Illumination Lab* (CIELAB) color space-based image analysis pipeline to quantify colorimetric signals. CIELAB calculates perceptually uniform color differences (ΔE) and thus reduces subjective bias compared to conventional RGB analysis. The signal enhancement protocol resulted in an assay limit of detection (LOD) of 34 CFU/ml in PBS buffer and 452 CFU/ml in spiked romaine lettuce without any enrichment. The composite nanoparticle labels have excellent stability and retain performance over one month of ambient storage. The reported LFIA assay addresses the sensitivity gap in conventional systems and demonstrates practical applicability for rapid, cost-effective, and field-ready monitoring of *L. monocytogenes* contamination in real food samples.

**Graphical abstract:**

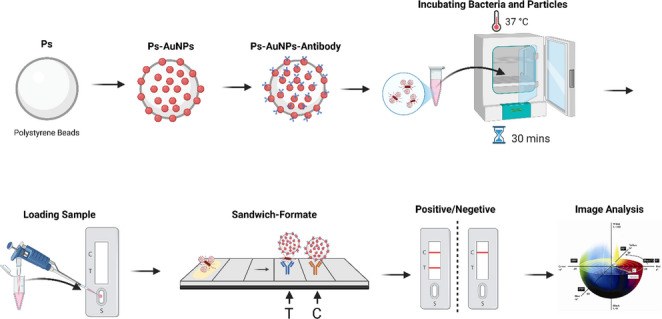

**Supplementary Information:**

The online version contains supplementary material available at 10.1007/s00604-025-07795-6.

## Introduction

*Listeria monocytogenes* is a saprophytic, Gram-positive bacterium and a significant foodborne pathogen responsible for listeriosis in humans and animals. This serious illness poses a considerable threat to public health and food safety, and it disproportionately affects vulnerable populations such as pregnant women, newborns, the elderly, and immunocompromised individuals. Listeriosis can lead to severe outcomes, including meningitis, septicemia, miscarriage, or neonatal death [1]. One of the biggest challenges in controlling *Listeria monocytogenes* is its remarkable ability to survive and grow under unfavorable conditions, such as low temperatures, high salt concentrations, and extreme pH levels. This adaptability allows it to persist as a contaminant in a wide variety of foods, ranging from fresh produce to ready-to-eat and frozen products [2]. The global burden of listeriosis has been increasing, especially in developed countries. For example, Europe has seen a significant rise in cases since 2008 compared to the 20th century [2]. To address these growing public-health concerns, researchers have explored a range of detection technologies, each with distinct advantages and disadvantages.

There are several conventional methods available for the detection of foodborne pathogens, such as culture-based microbiological techniques, nucleic acid-based methods, and immunological-based methods [3–6]. For *Listeria monocytogenes*, the standard culture-based microbiology techniques usually require 48–72 h to obtain results for negative controls and 72 h for positive controls [7]. For nucleic acid-based methods, specialized instruments are usually required when the experiments are performed, such as PCR (Polymerase chain reaction) nucleic acid amplification to analyze the gene sequences of *Listeria monocytogenes*. This usually takes a long time and exposes the experimenter to toxic reagents [7].

To address the conventional tool’s drawbacks, lateral flow immunoassays (LFIAs) emerged as promising tools because of their speed, portability, and ease of use [8]. Since most households used this type of test strip to detect new coronaviruses during the Covid-19 period, this is gradually becoming the most familiar to consumers as a home-based diagnostic method. These test strips are characterized by a short response time and no special environmental requirements for the application and can be easily operated in the field by personnel who do not require special training, which makes them very convenient [9]. Most LFIA are gold-nanoparticle (AuNP)-based and still suffer from low sensitivity and signal variability, which limits their applications in some cases.

Indeed, such AuNP based LFIA have been developed for *L. monocytogenes* detection [10]. However, despite notable improvements in sensitivity and specificity of these assays, they still face significant challenges and require further validation before routine use. Thus, comparatively few LFIA-based methods for *Listeria* are fully established or broadly implemented, especially relative to those for more frequently tested pathogens like *Salmonella* or *E. coli*. In practice, only a handful of commercial immunochromatographic kits target this pathogen in foods, which shows that this technology is underdeveloped for *L. monocytogenes* surveillance.

Moreover, because of *L. monocytogenes*’ typically low numbers in contaminated food and the need to detect live cells, most “rapid” *Listeria* tests are not truly point-of-care. Almost all current rapid test protocols include a selective enrichment step (often ~ 24 h) prior to the assay [11]. Even lateral flow strip tests for *Listeria* usually require culturing the sample in broth for 24–48 h to reach detectable levels [8]. For example, one review notes that in most commercial applications *Listeria* LFIAs are performed after enrichment, prolonging the total time to 1–2 days [8]. Although recent advances have shortened enrichment and improved sensitivity (yielding results in under ~ 8 h in the best cases) [8], there remains a scarcity of true point-of-care or on-site diagnostic tools that can rapidly detect *L. monocytogenes* directly in food samples. In contrast to pathogens like *E. coli* or viruses (for which self-contained rapid tests are common), *Listeria* still lacks widely available field-deployable detection kits.

On the other hand, most lateral flow assays for bacteria (including those targeting *Listeria*) have been designed as qualitative yes/no tests, interpreted by eye. Traditional strips do not require an instrument, but this also means results are subjective and merely presence/absence [12]. To overcome these limitations, researchers began to incorporate readers or smartphone-based apps to quantify LFIA signal intensity [12]. Notably, advanced color analysis techniques can significantly improve result interpretation by using methods such as CIELAB color space. The CIE Lab color space, standardized by the *International Commission on Illumination (CIE)* in 1976, is a perceptually uniform color model developed to closely represent human color perception based on the Opponent-Color Theory. This theory describes color vision as the result of opposing neural responses to pairs of colors (red versus green and blue versus yellow), along with a separate channel for lightness. This method is measuring precise color coordinates and improves consistency under different lighting [13]. Despite such progress in other applications, few *Listeria* LFIAs offer quantitative or colorimetric readouts. Most reported *Listeria* strips rely on naked-eye detection of a colored line, without objective measurement in color spaces like CIELAB. Automated or smartphone-based reading of *Listeria* test strips is still rare, which indicates an unmet need for quantitative approaches. Thus, to the best of our knowledge, current *Listeria* LFIA systems generally lack the kind of instrumentation or colorimetric analysis (e.g. digital color-value measurements) that can provide quantitative (intensity or concentration) rather than a simple binary result.

One solution to the issues related to the use of naked AuNPs is the use of polystyrene-gold composite nanoparticles (Ps-AuNPs). These preserve the excellent biocompatibility of gold nanoparticles while overcoming common drawbacks such as low sensitivity, poor color contrast, and nanoparticle aggregation that often limit traditional AuNP-based systems [14]. Similar Ps-AuNPs have previously been applied for detecting foodborne bacteria such as *E. coli* O157:H7 and *Salmonella Typhimurium* [14, 15], but they have not been applied to the detection of *Listeria Monocytogenes*. The combination of CIELAB color space analysis with the composite-labeling method, is likely to lead to an increase in sensitivity compared to the conventional Au-NPs reported in literature [16, 17]. Thus, the objective of this work is to demonstrate, for the first time, that a Ps-AuNP–based LFIA combined with CIELAB color analysis makes the rapid, quantitative, and enrichment-free detection of *Listeria monocytogenes* directly in food samples possible.

## Experimental section

### Materials synthesis

Ps-Au nanoparticles were obtained by using the citric acid reduction method using Gold (III) chloride trihydrate (HAuCl_4_·3H_2_O, ≥ 99.9%, Sigma-Aldrich, http://www.sigmaaldrich.com), Latex beads, mean diameter of 0.46 μm polystyrene (10% solid, 2 ml, LB5, Sigma-Aldrich) and Trisodium citrate dihydrate (USP testing specifications, Sigma-Aldrich) as raw materials. 100 µL original stock solution of Ps (polystyrene) particles were added to 20 ml of deionized water (DI) then stirred for 10 min at 300 rpm at room temperature. Subsequently, 26 ml of deionized water and 60 µl of 100 mg/ml Gold (III) chloride trihydrate (HAuCl4⋅3H2O, ≥ 99.9%) were added to the Ps solution and stirred for 5 min at room temperature (RT) with the stirring speed kept constant. The solution was placed on a rotating heating plate to increase the temperature from room temperature to 190 °C, keeping the stirring speed constant for 30 min. Then, 55 mg of trisodium citrate dihydrate was added and heating continued with stirring until the solution turned into a light pink color to stop stirring and heating. The non-homogeneous phase nucleation reaction occurred due to the large contact area provided by the polystyrene beads, leading to the formation of gold nanoparticles on the polystyrene surface. The fully reacted solution was allowed to cool down by placing it in a mixture of ice and water until the solution cooled down to room temperature, and then the solution was stored to 4 °C refrigerated and left to store in fridge overnight. The excess trisodium citrate dihydrate was removed by centrifugation (6500 rpm, 10 min) at 20 °C, and then the Ps-Au nanoparticles were resuspended in 0.01% TWEEN^®^ 20 solution (for molecular biology, viscous liquid) by vertexing. The optical density (OD) value of Ps-Au nanoparticle solution concentration was finally 0.45. Storage in a nonproteinic surfactant effectively prevents Ps-AuNP aggregation, which can be achieved for a long time.

### Antibody conjugation

*Listeria monocytogenes* Polyclonal Antibody (4–5 mg/ml, 1 mL, Polyclonal Rabbit IgG, PBS, pH 7.2, 0.1% sodium azide, Thermo Fisher Scientific, www.thermofisher.com) was conjugated with Ps-Au particles. Ps-Au nanoparticles stored in 0.01% TWEEN^®^ 20 solution were transferred to 1.5 ml centrifuge tubes (Eppendorf^®^ Safe-Lock microcentrifuge tubes, size 1.5 ml, PCR clean, polypropylene centrifuge tubes. Millipore Sigma, www.sigmaaldrich.com). Each centrifuge tube was filled with 1 ml of Ps-Au nanoparticle solution and centrifuged (6500 rpm, 10 min) at 20 °C. Resuspend the Ps-Au nanoparticles with deionized water so that the final concentration of the solution has an OD value of 1. Add *Listeria monocytogenes* Polyclonal Antibody to the Ps-Au nanoparticle solution at 50 µl of antibody in 1 mL and keep stirring at room temperature for one hour. BSA solution was added to give a final concentration of 0.25% and stirred for oner hour at room temperature, which serves to cover the unconjugated portion of the antibody from the Ps-Au nanoparticles and reduce non-specific adsorption. The solution was transferred to a centrifuge tube and centrifuged (6700 rpm, 10 min) and resuspended in a mixed solution (0.1 M PBS, 1% BSA, 0.25% Tween-20, 10% sucrose and 0.05% sodium azide) with a final concentration of OD 2.5 and stored in a fridge at 4 °C to keep the solution cold.

### Material characterization

Polystyrene and Ps-Au nanoparticles were imaged using a bright field transmission electron microscope (TEM), adjusted to a Tecnai G2 20 (Oxford Instruments) LaB6 filament, operating at 200 kV. The wavelength size of the Ps-AuNPs was obtained using a UV-visible spectroscopy with a Max Plus 384 spectrophotometer (Molecular Devices, Sunnyvale, CA, USA). Zeta potential measurements were performed using a Zetasizer Nano Z (Malvern Instruments Ltd. Westborough, MA, USA) to confirm gold reduction, antibody coupling and blocking processes [18]. The surface morphology and microstructure of nitrocellulose membranes were studied using scanning electron microscopy (SEM, Hitachi s-4800), especially for trapping Ps-AuNPs and bacteria, to confirm that LFIA was working.

### Lateral flow immunoassay strip fabrication and inkjet printer set up

Lateral flow test strips overlapped by 3 mm between each layer to facilitate the flow of sample from the sample pad to the absorbent pad. These strips were cut to 60 mm long and 3 mm wide. Compared to the Hi-flow 120 nitrocellulose membrane, the FF80 HP nitrocellulose membrane has better fluid transport properties and does not have excessive voids that would result in fluid flow onto the back pad, and thus the FF80 HP nitrocellulose membrane was chosen for subsequent experiments [15]. Laminated cards (60 mm) with FF80 HP nitrocellulose membrane (25 mm NC membrane), Whatman CF1, and Whatman CF5 dipstick pads were acquired from Cytiva.

Two types of nitrocellulose membranes with distinct capillary flow rates were evaluated during assay optimization. The primary difference between these membranes was the liquid flow speed, a parameter known to influence the immunoassay’s analytical performance. Membranes with slower capillary flow (longer flow times) allow increased residence time of antibody-labeled nanoparticle conjugates over the test line, which facilitate stronger antigen–antibody binding and thus, higher test line signal intensity. However, slower flow can also increase non-specific adsorption of labeled antibodies, contributing to background noise. In contrast, faster membranes reduce both overall assay time and non-specific binding but may lead to weaker test line signals due to insufficient interaction time.

The inkjet printing setup consists of two components: a syringe pump (ISPLab06, DK INFUSETEK) and an Automated Lateral Flow Reagent Dispenser (ALFRD, ClaremontBio). The power supply for the ALFRD operates at 120 VAC, providing an adjustable DC voltage ranging from 3 to 12 V. For this setup, the supply voltage connected to the ALFRD was set to 4.5 V. The syringe pump was configured with a total volume of 1000 µl and a flow rate of 0.2 ml/min. A 1 mL syringe (BD 1 ml Syringe, Seringue Luer-Lok™ Tip, Embout BD Luer-Lok™) was utilized in the setup. After configuring the inkjet printing parameters, two tubes of the ALFRD, containing the concentration of 0.5 mg/ml *Listeria monocytogenes* polyclonal antibody (4–5 mg/ml, 1 ml, Polyclonal Rabbit IgG, PBS, pH 7.2, 0.1% sodium azide, ThermoFisher Scientific, www.thermofisher.com) and 0.06 mg/ml Goat anti-Rabbit IgG (H + L) secondary antibody (Polyclonal IgG, ThermoFisher Scientific, www.thermofisher.com) as the printing materials, were used as syringes for the test line and control line, respectively. The distance between the test line and control line was set to 5 mm to prevent overlap when testing the mobile phase. Each line was printed in four overlapping layers. After printing, the strips were dried in a drying oven for at least 1 h, cut into individual strips 3 mm wide, and stored at dry room temperature.

In our tests, we monitored the signal intensity and non-specific binding on both candidate membranes by varying sample volume and observing the reproducibility of test line formation. We found that the membrane with moderate flow speed provided the best balance. This is yielding robust signals at the test line while minimizing background and avoiding the formation of irregularities due to membrane ‘voids’. These voids, where the nitrocellulose matrix is less densely formed, can create preferential fluid channels leading directly to the absorbent pad. This disrupts the uniform distribution of sample, reduces assay sensitivity, and contributes to inconsistent results. Thus, control of both membrane quality and capillary flow rate is necessary for optimal LFIA performance, as confirmed in our system.

### Bacteria culture preparation

The microorganisms tested in this work include the off-target *E. coli* O157:H7, *E. coli* K12, *Salmonella Typhimurium*, *Salmonella Enterica*, *Listeria innocua*, and the target, *Listeria monocytogenes*. Bacterial stock solutions, obtained from Purdue University’s Food Science Department, were stored frozen at -80 °C. Before further incubation, these bacterial stocks were grown overnight on agar plates. *E. coli* O157:H7, *E. coli* K12, and *Salmonella Typhimurium* were cultured in 30 ml of sterile Luria–Bertani (LB) media (Fisher Bioreagents, LB Broth, Lennox, Molecular Genetics Powder). *Salmonella Enterica* was cultured in 30 mL of Tryptic Soy Medium (BD Bacto™). *Listeria monocytogenes* and *Listeria innocua* were cultured in 30 mL of Brain Heart Infusion (BHI) media (BD Difco™). All bacterial cultures were incubated in a temperature-controlled orbital shaker at 37 °C and 160 rpm for 20 h. After growth, the bacteria were centrifuged once (6000 rpm for 10 min) and resuspended in 30 ml of 0.1 M PBS buffer (Sigma, Phosphate Buffered Saline, pH 7.4). Bacterial concentrations in the media were determined by selectively culturing E. coli strains on MacConkey Sorbitol Agar (BD Difco™), *Salmonella Typhimurium* on XLT-4 Agar (BD Difco™), *Salmonella Enterica* on *Tryptic Soy Agar* (BD Difco™), and Listeria strains on Modified Oxford Agar (BD Difco™).

### Lateral flow assay testing

*Listeria monocytogenes* was serially diluted in PBS buffer to concentrations ranging from 10¹ to 10⁷ CFU/ml. For each test, 20 µl of nanoparticle solution, 80 µl of mixture solution (comprising 0.1 M PBS, 1% BSA, 0.25% Tween-20, 10% sucrose, and 0.05% sodium azide), and 100 µL of the prepared bacterial solution were combined sequentially in a centrifuge tube. This mixture ratio was selected based on experimental optimization results. The tubes were incubated at 37 °C for 30 min. After incubation, the resulting mixture was applied to the sample pad of each lateral flow test strip, dried, rinsed with PBS buffer to remove non-specific interferences, and dried again before proceeding with image acquisition and analysis. To validate assay specificity, other bacterial species at a concentration of 10⁶ CFU·mL⁻¹ were tested following the same protocol. PBS buffer without bacterial solution was employed as a negative control. Each experimental condition was replicated three times for reproducibility. The immunochemical principle of the assay is described in Sect. "Detection mechaznism".

### Real sample testing

Romaine lettuce was selected as the real sample for this experiment. Fresh romaine lettuce was purchased from Walmart in West Lafayette and stored at 4 °C prior to use. For each experimental sample, two to three inner lettuce leaves were selected and weighed to obtain a total mass of approximately 25 g. Each sample was inoculated by evenly distributing 1 ml of a bacterial suspension containing 10⁷ CFU/ml of *Listeria monocytogenes* onto the leaf surfaces. After inoculation, samples were incubated in a biosafety cabinet at room temperature for 2 h to allow bacterial attachment. Subsequently, 225 ml of sterile PBS buffer (pH 7.4) was added to each sample, and the mixture was homogenized using a blender. The homogenized samples were transferred to sterile stomacher bags (710 ml, Whirl-Pak^®^ Homogenizer Blender Filter Bag) to filter out fibrous material and debris, yielding a clear solution. Serial dilutions of the filtered solutions were prepared to achieve target concentrations ranging from 10² to 10⁴ CFU/ml for lateral flow assays and spike recovery tests. Each experimental condition was repeated three times to verify reproducibility and reliability of the results.

### Image acquisition and CIELAB color space image analysis

The pictures of all the samples in this experiment were taken in an illuminated box, which provides stability of the light source for the captured images. The camera used to take pictures was a Nikon Z6 III camera with a Nikkor Z 24–120 mm/f4 zoom lens (1/500 sec, 100 mm/f4, ISO 100). All pictures were taken within 10 min of the end of the experiment.

**Fig. 1:**
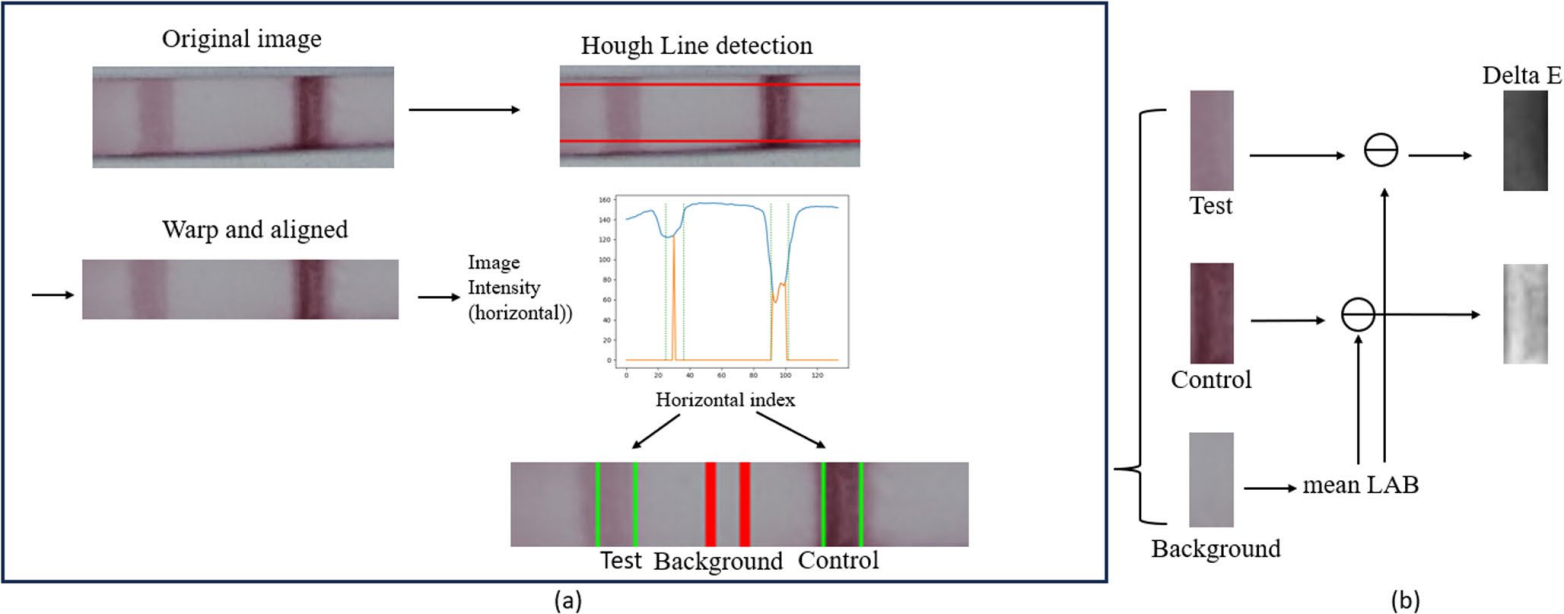
CIELAB color space analysis includes (**a**) image preprocessing pipeline: image calibration and the test/control band detection. (**b**) color extraction and Delta E calculation.

Each RGB picture contains multiple sample strips. We manually cropped out each strip and calibrated by detecting the boundaries of strips with Hough Line Transform [19]. Due to variations in lighting conditions and sample layouts, strip color differences may occur. Therefore, the intensity of test band is compared with the background region between test band and control band for each strip. We used CIELAB color space analysis [14] where the relative color intensity of test band is represented as $\:\varDelta\:E$ metric shown below (the full image processing workflow is illustrated in Fig. 1):

$$\begin{array}{lc}\triangle E\left(i,j\right)=\\\sqrt{\left(L\left(i,j\right)-L_{avg}\right)^2+\left(A\left(i\mathit,j\right)\mathit-A_{avg}\right)^2+\left(B\left(i\mathit,j\right)-B_{avg}\right)^2}\end{array}$$

The $\:i$ and $\:j$ are indexes of image pixels. The test band image and background image are separated into L, A and B three color channels. $\:\varDelta\:E$ represents the mean squared error between the average color of the background image ($\:{L}_{avg}$, $\:{A}_{avg}$ and $\:{B}_{avg}$) and per pixel color of the test band image ($\:L\left(i,j\right)$, $\:A\left(i,j\right)$ and $\:B\left(i,j\right)$). In Figs. 4, 5 and 6, “DeltaE” value is the average intensity of the ∆E image of the test band.

Figure 1 illustrates the process of CIELAB color space analysis [14]. In subfigure (a), the locations of test band and control band are identified by valley detection along horizontal axis after image calibration. In subfigure (**b**), Delta E images are calculated from LAB color extracted. The brightness of Delta E image is proportional to the intensity of color.

## Results and discussion

### Detection mechanism

The detection principle of the Ps-AuNP–based LFIA is based on the classic sandwich immunoassay in which antibody-functionalized Ps-AuNPs capture *Listeria monocytogenes* cells and migrate along the nitrocellulose membrane. At the test line, immobilized antibodies bind the Ps-AuNP-bacteria complexes, forming a visible pink band whose color intensity is proportional to bacterial concentration. Unbound conjugates continue to the control line where they are retained by secondary antibodies. The composite Ps-AuNP labels increase the optical contrast and reduce nonspecific aggregation compared with conventional AuNPs, resulting in an increased sensitivity. Because Ps-AuNP–bacteria complexes are larger than free nanoparticles, membrane flow rate and pore size were optimized to ensure complete migration, as discussed in Sect. "Bacteria culture preparation". Quantitative readout is achieved by computing the color difference (ΔE) in the CIELAB color space, which linearly correlates with the analyte concentration. This mechanism is at the basis of the assay’s enrichment-free and quantitative detection performance.

### Materials characterization

**Fig. 2:**
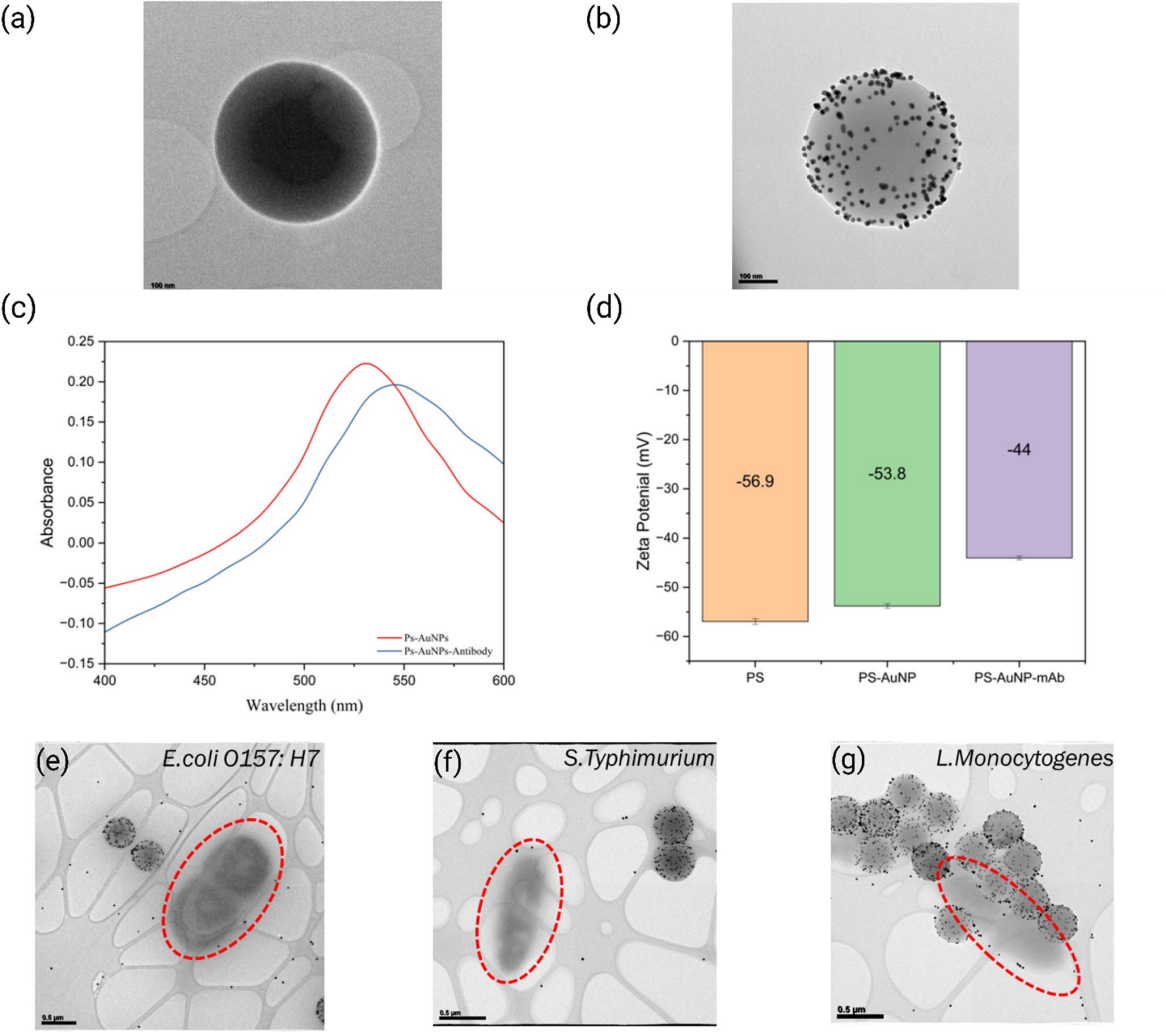
(**a**) TEM image of Ps beads. (**b**) TEM image of Ps-AuNPs. (**c**) UV–Vis spectra Ps-AuNPs and Ps-AuNPs-mAb. (**d**) Zeta-potentials of Ps, Ps-AuNPs, and Ps-AuNPs-mAb. The Ps-AuNP–mAb bar represents the zeta potential of particles after antibody conjugation and final BSA blocking. (**e**) TEM image antibody affinity testing for Ps-AuNPs-mAb incubated with *E. coli O157:H7.* (**f**) TEM image antibody affinity testing for Ps-AuNPs-mAb incubated with *Salmonella Typhimurium.* (**g**) TEM image antibody affinity testing for Ps-AuNPs-mAb incubated with *Listeria Monocytogenes*

AuNPs are commonly used in lateral flow tests due to their ease of preparation and excellent biocompatibility. However, AuNPs face challenges such as poor stability and a tendency to aggregate, which can negatively impact assay reliability and reduce color contrast. To address these issues, this study introduced a composite material that utilizes Ps as carriers to control the size and aggregation of AuNPs, as shown in Figs. 1a&b. The immobilization of AuNPs on polystyrene microparticles improves the color contrast in the mobile phase and overcomes the limitations of single colloidal gold, which results in improvements in both assay sensitivity and the stability of the detection material. The self-assembly of AuNPs on polystyrene particles was analyzed and confirmed using TEM, as shown in Figs. 2a & b.

To further confirm the successful modification of antibodies on gold nanoparticles, UV-visible spectroscopy was employed to analyze the peak shifts in the spectra of Ps-AuNPs and Ps-AuNPs-Antibody, as shown in Fig. 2c. The absorption peak of Ps-AuNPs was centered at a wavelength (λ_max_) of approximately 520 nm (Fig. 1c, red line), corresponding to the localized surface plasmon resonance (LSPR) band. After antibody modification, the spectrum has a red shift, with λ_max_ moving to 530 nm (Fig. 1c, blue line). This shift was attributed to changes in spatial site resistance caused by the presence of the modified antibody. These results are in good agreement with similar other literature reports, which gives us confidence in our successful functionalization of the nanoparticles [15, 20, 21].

The histograms in Fig. 2d show the changes in zeta potential of Ps beads following modification with AuNPs and subsequent antibody functionalization of the gold surface. The zeta potential increased after the deposition of gold nanoparticles onto the Ps beads and further increased after antibody modification on the gold nanoparticles. The zeta potential of PS particles was monitored at each stage of functionalization. Bare carboxylate PS particles exhibited a strong negative zeta potential due to surface carboxylate groups. Upon gold nanoparticle (AuNP) deposition, the surface charge shifted depending on the charge of the gold colloid, typically remaining negative due to citrate stabilization. Subsequent antibody conjugation reduced the magnitude of the negative charge, as proteins mask surface sites and introduce amino acid residues with charges near neutrality at physiological pH. Further blocking with BSA shifted the zeta potential closer to zero due to the dense protein layer on the particle surface. This is reflected in Fig. 2d, where the Ps-AuNP-mAb sample represents the particles after BSA blocking.

These results are in good agreement with results reported in the literature and confirm that Ps-AuNPs successfully achieved surface functionalization and are suitable for antigen detection [16]. This experiment also provides information on the band point properties and stability of the composite material.

### Optimization of testing conditions

To verify the smooth sample flow through the lateral flow test paper, the test solution must be carefully optimized. The concentration of the particulate solution was assessed by testing various optical density (OD) values, ranging from 0.5 to 2.3. Among these, the solution with an OD value of 1.0 demonstrated the best performance in terms of flow across the test paper. The concentration of the solution was measured using UV-visible spectroscopy, in Fig. 2c [15].

At the same time, the volume of antibody solutions was also optimized according to other literature reports. Specifically, it was found that a 50 ul volume of antibody added to 2 ml volume of Ps-AuNPs solution led to best results [22]. At the same time, the addition of 50 ul of 10% BSA solution for covering, effectively prevents the Ps-AuNPs from reacting with other non-specific antigens, to achieve the best results of the detection.

To verify that the composite can effectively capture *Listeria monocytogenes* after antibody modification, transmission electron microscopy (TEM) was used to confirm the results (Fig. 2e and f, and 2g). Sample preparation involved mixing the Ps-AuNPs-Antibody particulate solution with *E. coli O157:H7*, *Salmonella Typhimurium*, and *Listeria monocytogenes* in a 3:1 volume ratio at a concentration of 10^7^ CFU/ml, followed by incubation for 20 min. A 5 µl aliquot of the mixed solution was deposited onto a TEM grid, dried, and then imaged using TEM. The TEM images, as shown in Fig. 2g, revealed strong affinity binding of the antibody to *Listeria monocytogenes*, with the particles showing optimal coverage around its cells. In contrast, no nonspecific interactions were observed between the antibodies and the surfaces of *E. coli O157:H7* or *Salmonella Typhimurium*, as shown in Fig. 2e & f. These conditions were based on prior optimization of conjugate-to-bacteria volume ratio reported in our earlier Ps-AuNP LFIA study.

### Optimization of inkjet printing platform

The inkjet printing platform was assembled using an Automated Lateral Flow Reagent Dispenser (ALFRD) and a syringe pump, both purchased from the manufacturer. After connecting the ALFRD and syringe pump, a semi-automatic inkjet printing system was successfully constructed. To optimize performance, the voltage of the device and the flow rate of the syringe pump were continuously adjusted to allow the antibody solution to pass through the inkjet printing platform and produce a uniform, straight line on the test paper without dispersing. Simultaneously, the antibody concentration was also optimized.

Through repeated experiments, it was determined that diluting the antibody solution 10-fold yielded the most effective results, with minimal dispersion observed when multi-layer lines were printed. The voltage of the inkjet printing machine was tested across a range of 3.0 V to 4.5 V, and it was found that the platform operated most stably at 4.5 V. At this voltage, further testing of the syringe pump revealed that a flow rate of 0.2 ml/min produced optimal results. The final printed lines on test paper are shown in Figure Aa & C (Supplementary Information).

### Microstructure analysis of the antibody-based lateral flow test

**Fig. 3:**
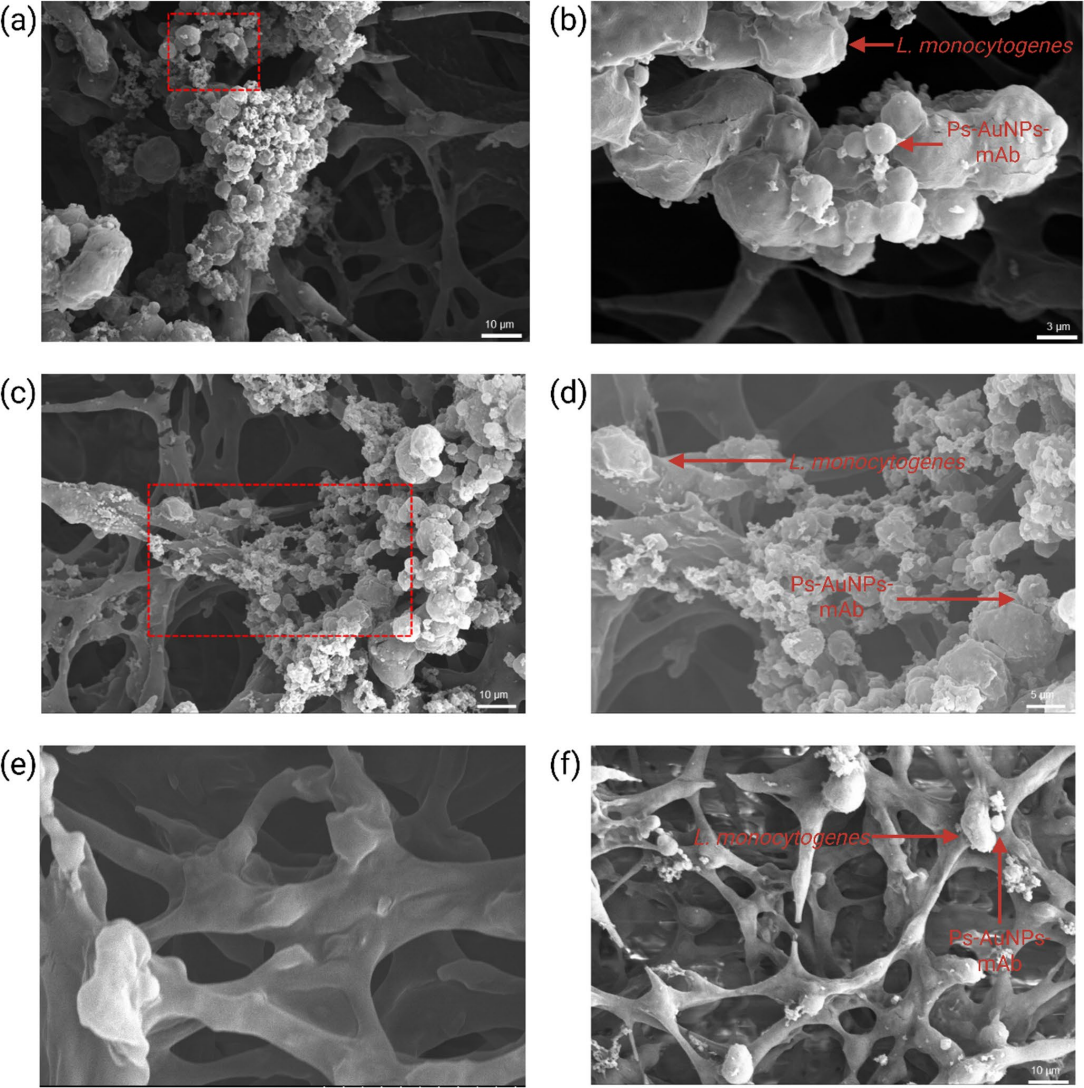
(**a**) Top-view of SEM images of Ps-AuNPs-mAb with *Listeria monocytogenes* on the test line. (**b**) The zoom-in image of the marked area in **a**. The arrows in the images show the bacteria and particles present on the test line. (**c**) Top-view of SEM images of Ps-AuNPs-mAb with *Listeria monocytogenes* on the control line. (**d**) The zoom-in image of the marked area in **c**. The arrows in the images show the bacteria and particles present on the control line. (**e**) Original NC membrane morphology. (**f**) *Listeria monocytogenes* and Ps-AuNPs-mAb on NC membrane after sample flow through LFIA test strip. The arrows in the images show the bacteria and particles present in the NC membrane

Top-view scanning electron microscopy (SEM) was employed to observe the capture of *Listeria monocytogenes* and the flow of particles within the LFIA strips. After standard sample preparation, the LFIA strips were dried 10 min before the examination. The comparison of surface morphology and NC membranes with and without *Listeria monocytogenes* is presented in Fig. 3. SEM micrographs (Fig. 3a & c) clearly reveal that antibodies successfully captured *Listeria monocytogenes* in both the test line and the control line regions. Additionally, a comparison of other regions on the NC membrane before and after sample addition showed significant differences. In the absence of *Listeria monocytogenes*, the original NC membrane morphology displayed large voids, as shown in Fig. 3e. Conversely, upon the addition of bacteria, aggregation occurred, with particles trapped on the fibers of the NC membrane, filling the voids with numerous attached particles (Fig. 3f). An energy-dispersive X-ray spectroscopy (EDS) analysis was performed to evaluate the deposition of Ps-AuNPs-mAb complexes by averaging measurements from six selected points within the provided figure. The results indicated an atomic percentage (at%) of gold (Au) at 2.21%, as shown in Figure Bb in the Supplementary Information. This confirms that the material remained stable during testing and that gold was uniformly distributed on the surface of polystyrene beads.

### Specificity and sensitivity testing in buffer solutions

**Fig. 4:**
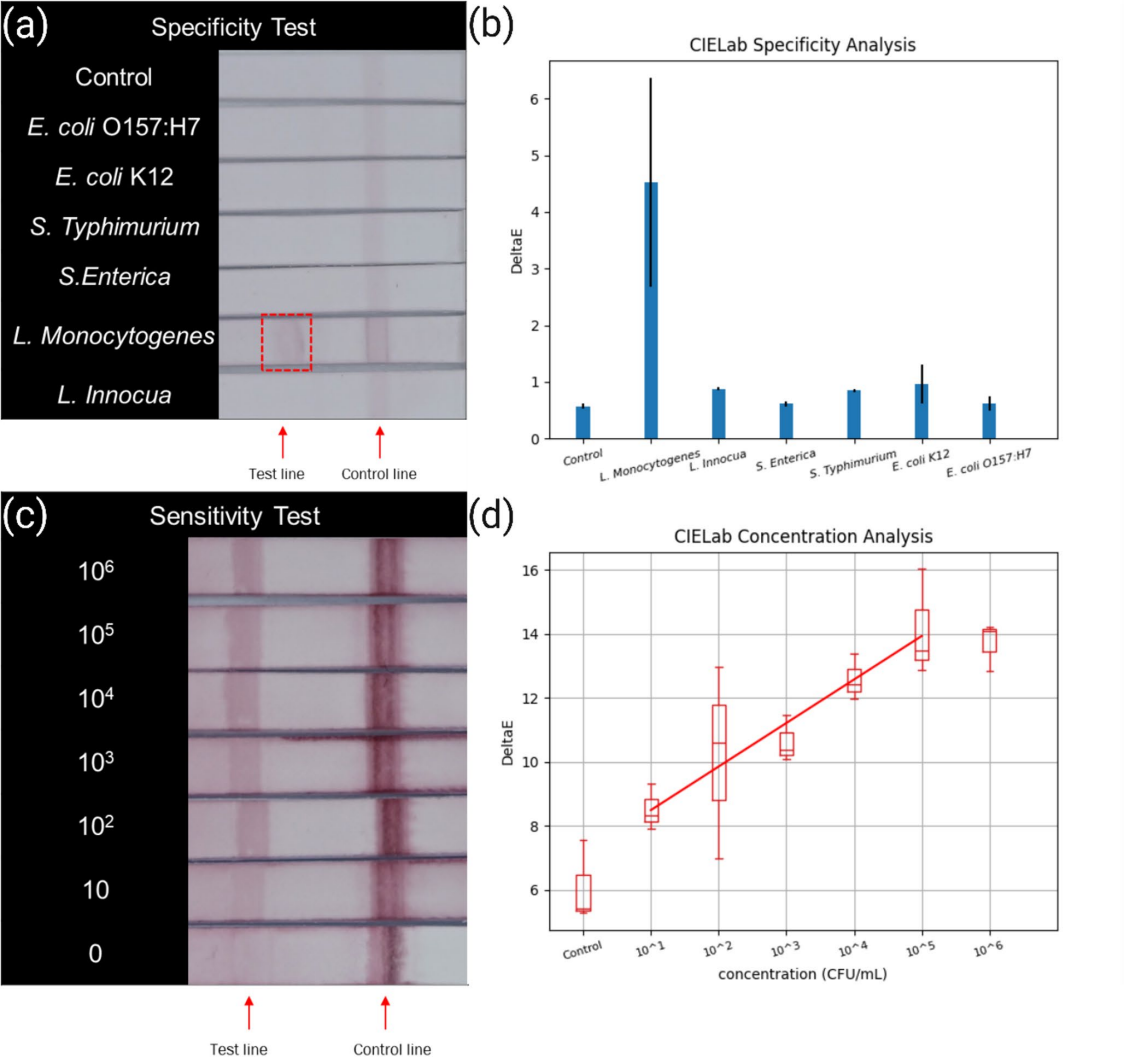
Specificity and sensitivity test in PBS. (**a**) All bacteria were tested at 10^5^ CFU/ml for specificity test color image. Only *Listeria Monocytogenes* results show positive. (**b**) The CIELAB color image analysis results show *Listeria Monocytogenes* the highest signal. (**c**) The color image shows *Listeria Monocytogenes* sensitivity test in 0 to 10^6^ CFU/ml. (**d**) The CIELAB color image analysis results show the linear of *Listeria Monocytogenes* concentration 0 to 10^6^ CFU/ml signal

To validate the specificity of the LFIA platform we developed, several of the off-target common food-contaminating bacteria and one non-pathogenic *Listeria* strain were selected for testing in PBS buffer: *E. coli* O157:H7, *E. coli* K12, *Salmonella Typhimurium*, *Salmonella Enterica*, *Listeria monocytogenes*, and *Listeria innocua*. All bacterial samples were prepared at a concentration of 10^5^ CFU/ml in PBS buffer. The results were evaluated based on visual assessment of color intensity on the test line and CIELAB image analysis. Images were captured 20 min after drying the test paper (as shown in Fig. 4a), which displayed the original color bands of the ABLF test. Among the tested bacteria, *Listeria monocytogenes* showed a clearly positive reaction with a distinct color response in the test area. CIELAB analysis (Fig. 4b) confirmed that *Listeria monocytogenes* produced the highest peak, indicating the strongest reaction compared to the other five bacterial strains.

The sensitivity of the *Listeria monocytogenes* LFIA was evaluated through serial dilution over a concentration range of 0 to 10^6^ CFU/ml, as illustrated in Fig. 4c. The results from CIELAB image analysis (Fig. 4d) demonstrated a strong linear correlation between color intensity and bacterial concentration within the range of 0 to 10^5^ CFU/ml, with a correlation coefficient of 0.928. This result is in good agreement with previous studies, which showed that bacterial concentrations exceeding 10^5^ CFU/ml can lead to diminished color intensity due to increased nonspecific binding at higher concentrations [15]. In our study, the detection limit of the LFIA was found to be 34 CFU/ml in PBS. The detection limit was defined as the lowest bacterial concentration yielding a visually discernible test line and a ΔE signal greater than three standard deviations above the negative control, with concentrations verified by plate counting on selective agar.

Although the visual intensity of the test line at high bacterial concentrations may appear modest under ambient lighting, quantitative CIELAB analysis yields a strong and linear ΔE response. This demonstrates that the platform’s main advantage lies in its quantitative detection capability rather than subjective visual contrast. Minor variation in control line width was occasionally observed due to the inkjet deposition process, but all strips exhibited functional control lines, which is a confirmation of the correct assay flow and reagent activity.

### Specificity and sensitivity testing in Romaine lettuce

**Fig. 5:**
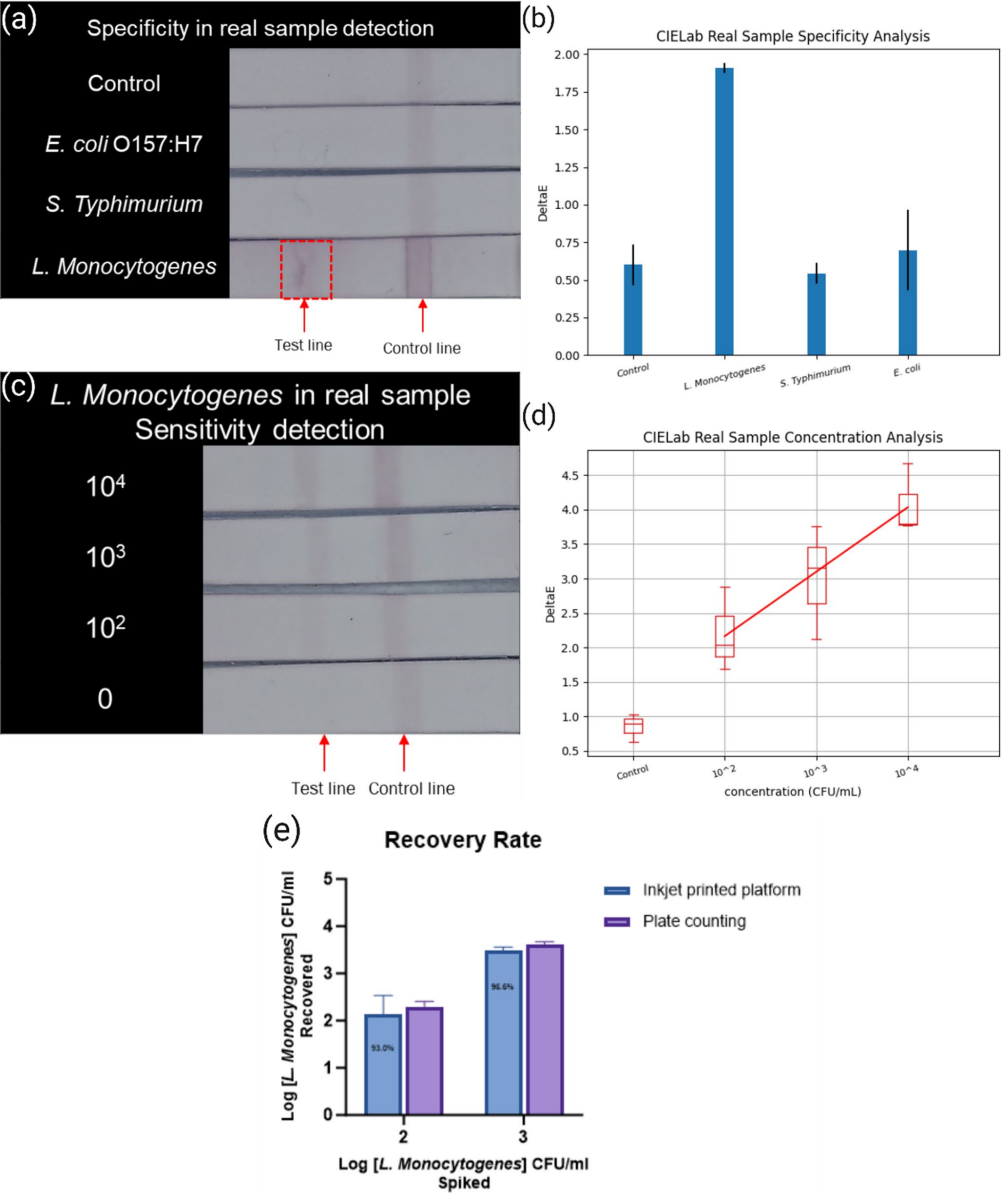
Specificity and sensitivity test in romaine lettuce. (**a**) Color image result of three different bacteria in 10^4^ CFU/ml. (**b**) The CIELAB color image analysis result shows the highest signal for *Listeria monocytogenes*. (**c**) Color image result of sensitivity test for Listeria monocytogenes concentration from 0 to 10^4^ CFU/ml. (**d**) The CIELAB color image analysis results show the linear of *Listeria Monocytogenes* concentration 0 to 10^4^ CFU/ml signal. (**e**) Recovery performance of the proposed platform for detection of *L. monocytogenes* from 10^2^ to 10^3^ CFU/ml

To validate the practical application of our LFIA platform for detecting *Listeria monocytogenes*, we simulated a real-life complex environment by introducing the bacterium into Romaine lettuce samples. After multiple rounds of testing, the platform demonstrated a linear range of 10^2^ ~ 10^4^ CFU/ml, and a detection limit of 10^2^ CFU/ml, as illustrated in Fig. 5d. This LOD of 10^2^ CFU/ml in real complex food samples represents a significant improvement compared to previous methods (See Table 1) [23–27]. For example, Shi, L. et al. reported a detection limit of 10^4^ CFU/ml in Chicken, Fish, Flammulina velutipes, etc [23]. The improved sensitivity achieved with our newly developed composite material is a significant improvement in food safety testing of *Listeria monocytogenes* in particular. Additionally, CIELAB image analysis revealed that the obtained Δ*E* values were linear, with a correlation coefficient of 1.000. This shows a strong positive correlation between bacterial concentration and signal intensity, in good agreement with established detection trends and corroborating findings from other studies [14, 15]. We tested the specificity of the LFIA assay against nontarget pathogens *Escherichia coli* O157:H7, and *Salmonella Typhimurium*, at concentrations of 10^4^ CFU/ml. As shown in Fig. 5a, *Listeria monocytogenes* had the strongest signal intensity during CIELAB image analysis, which represents highly significant positive results. This shows the specificity of our detection platform for *Listeria monocytogenes* is holding up in complex food samples, as well as in buffer solutions.

To more accurately quantify the specific concentration of *Listeria monocytogenes*, we re-validated the results obtained from the ABLF platform assay using traditional plate counting methods. As shown in Fig. 5e, the recoveries ranged from 93.0% ~ 96.6% when the pathogen concentration was between 10^2^ and 10^3^ CFU/ml, which indicates high accuracy. The significantly lower signal intensities observed in lettuce samples compared to PBS at the same Listeria concentrations are attributed to matrix effects inherent to complex food extracts. These effects include increased viscosity, non-specific adsorption of matrix macromolecules onto the membrane or detection antibodies, and the presence of interfering plant components that can inhibit antibody-antigen binding. These factors reduce assay sensitivity because they impend target capture and signal generation, as consistently reported in previous LFIA studies comparing buffer and food matrices [28–30]. This validation demonstrates the effectiveness of the inkjet printing system in depositing antibody inks into nitrocellulose membranes, along with the preservation of test stability even in the presence of food matrix interferences.

Statistical comparisons with other reported detection methods, summarized in Table 1, show the superior sensitivity of our platform. Utilizing Ps-AuNPs-mAb, a composite material previously reported by our lab for the detection of *E. Coli* O157:H7 [14], our LFIA test paper achieves a limit of detection as low as 34 CFU/ml in PBS buffer solution and 452 CFU/ml in complex food matrices. Only colorimetric LFIAs are listed to provide a consistent comparison with the present study; more recent reports often use fluorescence or electrochemical formats that are not directly comparable.

**Table 1:** An overview of recently reported lateral flow assays for determination of *Listeria monocytogenes*

Analytical method	Probe signal for using materials	LOD in PBS buffer (CFU/ml)	LOD in food sample (CFU/ml)	Food sample	Ref.
Colorimetric LFA	^a^HRP-SHP-30	95	97	Reduced fat milk	[24]
Colorimetric LFA	AuNPs	None	3.7 × 10^6^	Milk	[25]
Nucleic acid Colorimetric LFA	^b^CNPs	10^5^	10^5^	Milk	[26]
Colorimetric LFA	^c^CFSMP	10^4^	10^4^	Chicken, Fish, Flammulina velutipes, etc.	[23]
Colorimetric LFA	^d^AuNP-CB	10	10	Pork	[27]
Colorimetric LFA	**Ps-AuNPs**	**34**	**452**	**Romaine lettuce**	**This work**

^a^HRP-SHP-30, Horseradish peroxidase with Iron oxide (Fe_3_O_4_) magnetic particle

^b^CNPs, Carbon nanoparticles

^c^CFSMP, Carboxyl-functionalized superparamagnetic particles

^d^AuNP-CB, Cucurbit- functionalized gold nanoparticles

It is relevant to discuss these results in the context of other methods, such as magnetic bead-based pretreatment. This is a method that is widely used to improve LFIA performance in complex foods by: (i) concentrating targets from larger sample volumes and (ii) removing a portion of interfering matrix components before detection. Recent reports [27] show that MNP capture can lower apparent LODs and improve robustness in challenging matrices. However, these benefits come with some practical issues. MNP methods introduce additional steps, including incubation with beads, magnetic separation, and often bead washes, require magnet hardware and bead functionalization, increase per-test consumable cost, and extend total time-to-result. By comparison, the Ps-AuNP LFIA reported in the current work, comes with an easier, enrichment-free protocol. At the same time, it delivers quantitative, on-strip readout. The method achieved a LODs of 34 CFU/mL in PBS and 452 CFU/mL in romaine lettuce without magnetic capture or culture enrichment, with same-day results and facile on-site usability. Thus, while MNP pretreatment can further improve sensitivity, our results show that engineered colorimetric labels combined with CIELAB analysis can reach performance that is competitive with many MNP-assisted assays, with lower procedural complexity and equipment burden. These results establish our platform as one of the few reported detection systems for *Listeria monocytogenes*, capable of maintaining high sensitivity under challenging conditions, without an enrichment step (see Table 1).

### Sensor stability

**Fig. 6:**
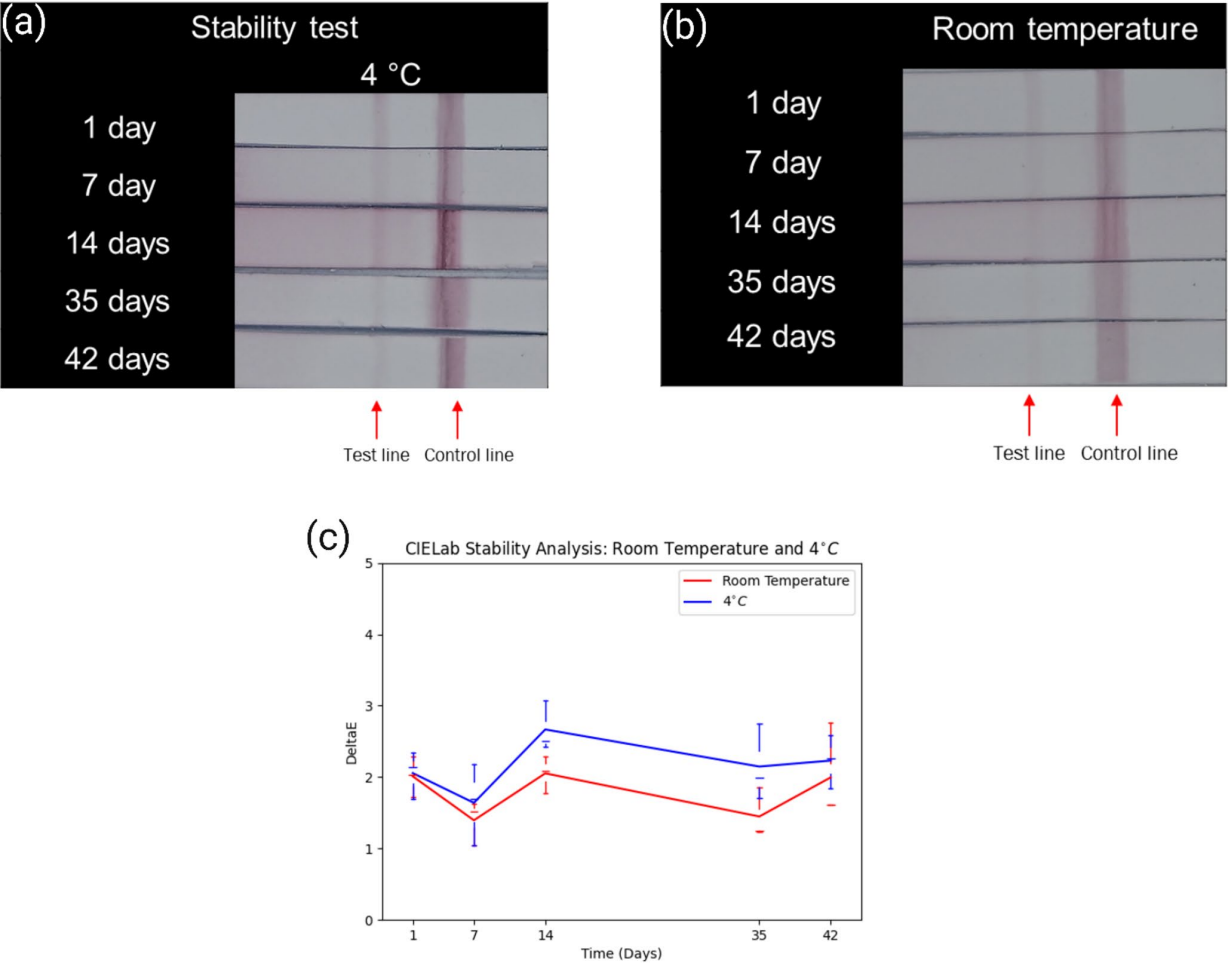
Stability test. (**a**) Color images for 1,7,14,35 and 42 days at 4 °C storing strips. (**b**) Color images for 1,7,14,35 and 42 days at room temperature storing strips. (**c**) CIELAB Color Analysis of ABLFL Stability from Day 1 to Day 42 at Two Different Storage Temperatures

To evaluate sensor performance under real-world storage and transportation conditions, we conducted stability tests over 42 days at two storage temperatures (room temperature and 4°C refrigeration), with assessments at intervals of 1, 7, 14, 35, and 42 days, as shown in Fig. 6a&b. The test solution used 10^4^ CFU/ml *Listeria Monocytogenes* solution which is in romaine lettuce. Triplicate measurements are performed for reproducibility. In Fig. 6c, image analysis revealed refrigerated (4°C) LFIA test strips maintained superior stability compared to ambient-stored counterparts. The observed accuracy decline at room temperature aligns with literature reports suggesting partial antibody degradation under non-refrigerated conditions [17]. Nevertheless, detection performance remained remarkably stable throughout the 42-day period under both storage regimes, demonstrating the system’s applicability for practical deployment.

## Conclusion

This study presents a lateral flow immunoassay for the detection of *Listeria monocytogenes* using a composite nanoparticle system, Ps-AuNPs-mAb, that improves sensitivity, improves visual clarity, and reduces nonspecific aggregation compared to conventional gold nanoparticle systems. The assay provides a quantitative readout that yields consistent, linear responses across both buffer solutions and real food samples via CIELAB color space analysis. This assay operates without an enrichment step. Thus, it allows direct application to food matrices and makes same-day results possible. In romaine lettuce, the LOD reached 452 CFU/ml, and in PBS buffer, as low as 34 CFU/ml. These results confirm the assay’s strong sensitivity and selectivity in both controlled and complex conditions.

The detection strips remained stable over an extended period and performed reliably under ambient storage conditions, which renders this sensor practical for field testing. Overall, the platform developed in this work offers a rapid, quantitative, and user-accessible diagnostic tool for food safety monitoring, especially suited to applications where fast and accurate detection of *L. monocytogenes* is essential.

## Supplementary Information

Below is the link to the electronic supplementary material.

Supplementary Material 1

## Data Availability

No datasets were generated or analysed during the current study.
