# Supplementary material for: Rapid, quantitative lateral flow immunoassay using polystyrene-gold composite nanoparticles and CIELAB analysis for on-site detection of Listeria monocytogenes in food samples
Source: Mikrochim Acta. 2025 Dec 28;193(1):50. doi: 10.1007/s00604-025-07795-6 (PMC12743664; doi:10.1007/s00604-025-07795-6)
Supplement: Supplementary file 1 — Supplementary Material 1 [file 604_2025_7795_MOESM1_ESM.docx]

**Supplementary Information (SI)**

**Rapid, Quantitative Lateral Flow Immunoassay Using Polystyrene-Gold Composite Nanoparticles and CIELAB Analysis for On-site Detection of Listeria monocytogenes in Food Samples**

Zhijian Wang^1^, Ya-Ching Yu^2^, Xiaoyu Ji^3^, Yixuan Ding^2^, Amanda J. Deering^5^, George T.-C. Chiu^4^, Jan P. Allebach^3^, Lia A. Stanciu^1,2,6^*

^1^ Weldon School of Biomedical Engineering, Purdue University, 610 Purdue Mall, West Lafayette, IN, 47907, USA

^2^School of Materials Engineering, Purdue University, 701 West Stadium Ave., West Lafayette, IN, 47907, USA

^3^School of Electrical and Computer Engineering, Purdue University, 465 Northwestern Ave, West Lafayette, IN, 47907, USA

^4^School of Mechanical Engineering, Purdue University, 585 Purdue Mall, West Lafayette, IN, 47907, USA

^5^Department of Food Science, Purdue University, 745 Agriculture Mall Dr, West Lafayette, IN, 7907, USA

^6^Bindley Bioscience Center, Purdue University, 1203 W State St, West Lafayette, IN, 47907, USA

* Corresponding author. Department of Materials Engineering, Purdue University, West Lafayette, IN, 47907, USA.

E-mail address: lstanciu@purdue.edu (Lia Stanciu).

Phone number: 765-496-3552

**
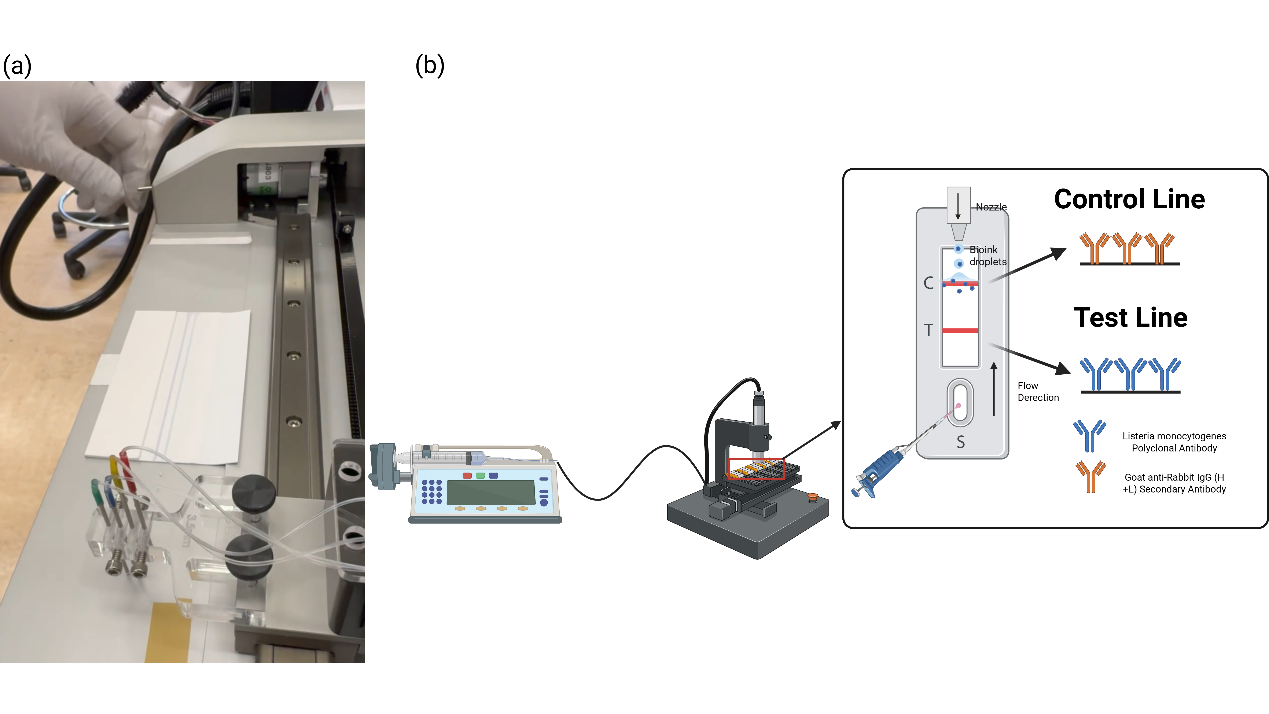
**

**Figure A.** (a) Inkjet prints the real image. The nozzle will move from left to right, dropping bio-ink at each position to print the test and control lines on the NC membrane. (b) Inkjet printing for ABLF construction. (Created with BioRender.com)

**
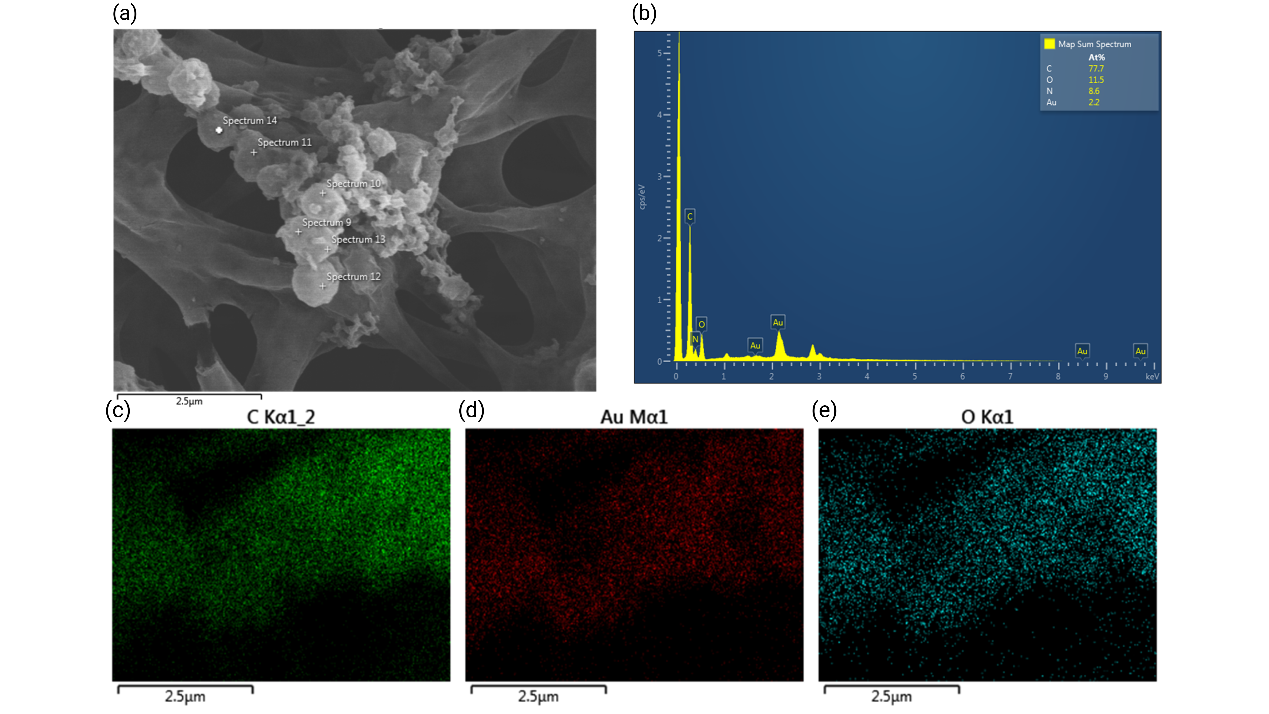
**

**Figure B**. EDS analysis of (a) the image on the control line region with six identified points, (b) quantification results in atomic ratio and elemental spectra. (c) EDS analysis of Carbon atomic image. (d) EDS analysis of Gold atomic image. (e) EDS analysis of Oxygen atomic image.

**
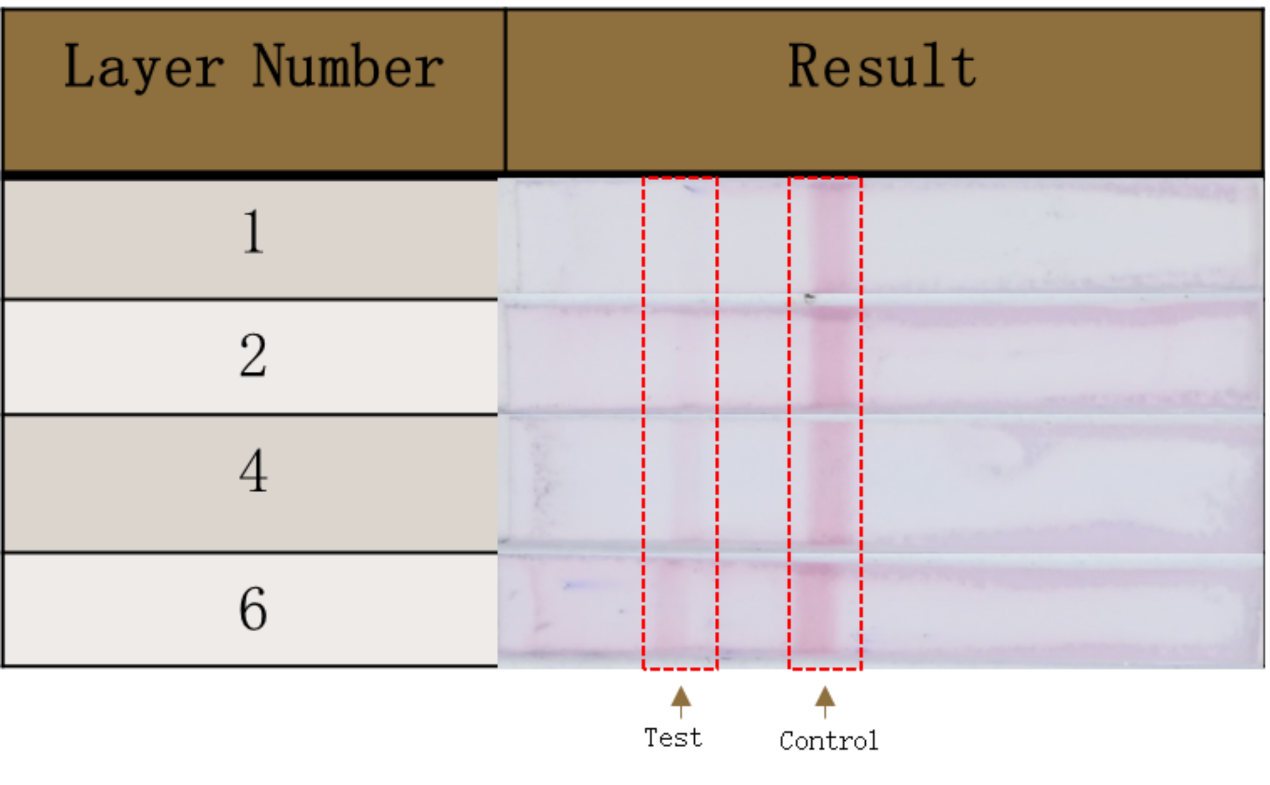
**

**Figure C.** Optimized printing antibody layer.
